# Exploring the Application of Graphene Oxide-Based Nanomaterials in the Repair of Osteoporotic Fractures

**DOI:** 10.3390/nano14060553

**Published:** 2024-03-21

**Authors:** Hongfa Zhou, Jinyuan Chen, Xuan Zhang, JingJing Chen, Jiayou Chen, Shicheng Jia, Deli Wang, Hui Zeng, Jian Weng, Fei Yu

**Affiliations:** 1 Department of Bone & Joint Surgery, Peking University Shenzhen Hospital, Shenzhen 518036, China; 18hfzhou@stu.edu.cn (H.Z.); cjyoffice2000@163.com (J.C.); 19xzhang@stu.edu.cn (X.Z.); wangdelinavy@163.com (D.W.); 2 National & Local Joint Engineering Research Center of Orthopaedic Biomaterials, Shenzhen 518036, China; 3 Shenzhen Key Laboratory of Orthopaedic Diseases and Biomaterials Research, Shenzhen 518036, China; 4 Medical College, Shantou University, Shantou 515063, China; cjj1417666324@163.com (J.C.); chenjy510137@gmail.com (J.C.); jessejia0910@gmail.com (S.J.); 5 Department of Orthopedics, Shenzhen Second People’s Hospital, Shenzhen 518000, China; zenghui_36@163.com

**Keywords:** graphene oxide, nanomaterial, osteoporotic fractures

## Abstract

Osteoporotic fractures are induced by osteoporosis, which may lead to the degradation of bone tissues and microstructures and impair their healing ability. Conventional internal fixation therapies are ineffective in the treatment of osteoporotic fractures. Hence, developing tissue engineering materials is crucial for repairing osteoporotic fractures. It has been demonstrated that nanomaterials, particularly graphene oxide (GO), possess unique advantages in tissue engineering due to their excellent biocompatibility, mechanical properties, and osteoinductive abilities. Based on that, GO-nanocomposites have garnered significant attention and hold promising prospects for bone repair applications. This paper provides a comprehensive insight into the properties of GO, preparation methods for nanocomposites, advantages of these materials, and relevant mechanisms for osteoporotic fracture applications.

## 1. Introduction

**Figure 1:**
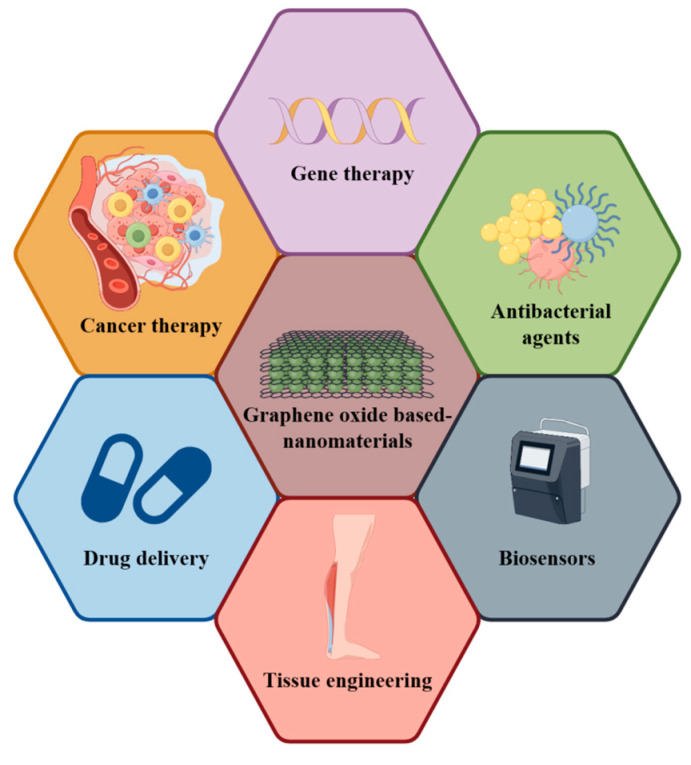
Application of GO-based nanomaterials.

Osteoporosis, a systemic skeletal disease, is pathologically characterized by a decrease in bone mass and the deterioration of bone tissue microstructure. These changes result in bone fragility and susceptibility to fractures. The disease is mainly manifested as the degeneration of the bone mass and bone microstructures, which may exert adverse impacts on fracture treatment [1]. Fractures resulting from osteoporosis predominantly occur in women aged over 55 years and men over 65 years [2]. These fractures contribute to the morbidity of other skeletal disorders, thereby increasing mortality rates and healthcare expenditures [2]. Each year, osteoporosis affects approximately nine million people worldwide [3], with the total healthcare costs associated with osteoporotic fractures estimated to be around USD 4.5 billion annually [4]. Currently, osteoporotic fractures are mainly treated using conservative methods, such as casts or orthotics, or the implantation of fixations like screws and plates [5]. However, there are several challenges in the treatment of these fractures [6]. Primarily, osteoporosis leads to reduced bone density and increased bone fragility. Consequently, patients with osteoporosis often experience fractures manifested as more and smaller fragments, leading to more complex fractures than those of healthy bones [7]. These complex fractures require superior surgical skills for repositioning and more effective implantation of fixations to stabilize the fracture ends. Secondly, these patients are prone to falling, which escalates over time [8]. This can be attributed to a decline in balance, proprioception, and visual acuity with age. Furthermore, the weight-bearing capacity in these patients tends to be lower compared with those with non-osteoporotic fractures, thus resulting in higher overloading risks of internal fixation [9]. Finally, the mechanical properties of the osteoporotic bone are compromised due to the significant loss of bone mass, which is primarily reflected in the porous cancellous and thin cortical bone and the poor load-bearing capacity of implants [10]. As a result, complications such as endoprosthetic loosening, rupture, and peripheral fractures are common in osteoporotic fractures [11]. Given these challenges, there is an urgent demand for materials that can be employed to stabilize the fracture and promote bone production. Nanotechnology is an interdisciplinary field involving medicine, biology, chemistry, engineering, materials science, and physics. The advent of nanotechnology in medicine has empowered scientists to engineer novel materials [12]. On the nano scale, materials exhibit significant alterations in their chemical, physical, and biological properties. Nanomaterials present some unique advantages. For instance, some nanomaterials bear structural similarities to natural bone and possess excellent mechanical properties, such as desirable electrochemical capacity, large specific surface area, and superior wettability. These characteristics facilitate cellular support while regulating the proliferation, differentiation, and migration of cells, ultimately enhancing bone repair effects [13]. Meanwhile, graphene family materials are used in bone tissue engineering, such as in vivo mediating cellular osteogenic differentiation and promoting bone regeneration, and can also be added as reinforcement materials in scaffolds to enhance mechanical properties and improve physicochemical characterization [14]. As a graphene derivative, graphene oxide (GO) can be prepared through the chemical oxidation of graphite flakes in potent oxidizing agents. It is a quintessential two-dimensional nanomaterial [15]. Owing to the unique properties of GO, GO-based nanocomposites have garnered considerable attention in various biomedical fields, such as drug/gene delivery, biosensing, cancer therapy, and tissue-engineered scaffolds (Figure 1) [16]. More importantly, GO is also an excellent choice for use in bone tissue engineering due to its remarkable specificity, chemical stability, and compatibility with biological systems [17]. In this paper, the potential and advantages of GO-based nanocomposites in osteoporotic fractures will be systematically outlined. Additionally, some limitations in the application of this material will be highlighted with pertinent recommendations.

### An Introduction to GO and GO-Based Nanomaterials

The preparation methods of GO are primarily divided into three categories: the Brodie method, the Staudenmaier method, and the Hummers method [18]. However, the Staudenmaier method and the Brodie method have certain drawbacks. They produce harmful gases like ClO_2_ and NO_2_ during the reaction process, require a significant amount of time, and consume excessive raw materials when used under prolonged reaction conditions [19]. As a result, the Hummers method is generally preferred as the preparation method for GO. The Hummers method offers several advantages compared to previous technologies. Firstly, the reaction can be completed within a few hours. Secondly, by replacing KClO_3_ with KMnO_4_, the reaction safety is improved, and the risk of explosive ClO_2_ precipitation is avoided. Thirdly, NaNO_3_ is used instead of HNO_3_, eliminating the formation of acid mist [20]. However, the Hummers method still has some room for improvement. It often results in excessive acid residue that is challenging to remove, and the oxidation degree of GO may not be sufficient [21]. Consequently, many researchers have made various improvements to the preparation of GO using the Hummers method.

As a promising material in biotechnology, GO has a structure comprising a two-dimensional honeycomb lattice of carbon atoms and numerous oxygen-containing groups, such as carboxyl, hydroxyl, and epoxy groups [22]. These groups are modified at the basal surface and edges. GO not only inherits the superior mechanical properties and large specific surface area of graphene but also exhibits excellent hydrophilicity and bioactivity due to the abundance of oxygen-containing groups on its surface [23]. Moreover, GO can be easily dispersed in water and various organic solvents, forming stable dispersions containing one or a few layers of single-atom layer structures. These structures can self-assemble with other materials, leveraging the amphiphilicity and layered structure of GO. Alternatively, other materials can be introduced onto the surface of GO or between the layers. Then, these materials grow on the GO substrate through chemical or thermal reactions, forming uniform GO-based nanocomposites [23,24]. GO also has good antibacterial properties, and it has been shown that it can degrade bacterial cell membranes by releasing large amounts of adenine and proteins on the bacterial surface [25].

**Figure 2:**
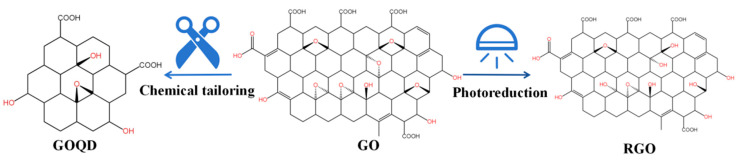
Structural formula of GO and its derivative.

GO can also be chemically or physically prepared as GO derivatives (Figure 2), such as Graphene Oxide Quantum Dots (GOQDs) or Reduced Graphene Oxide (RGO). More specifically, GOQDs can be prepared via electrochemical oxidation [26,27], hydrothermal treatment [28,29], and microwave-assisted reaction or via other physicochemical processes to selectively sever the carbon–carbon bond of GO [30]; RGO can be prepared through chemical vapor deposition [30,31,32], laser reduction, the flash lamp photothermal method [33,34], and other methods to reduce the oxygen content of GO, with the aim of removing the oxygen-containing groups on GO and restoring the conjugated structure [35]. These derivatives not only inherit many functional properties of GO but also possess their own unique functions, such as the tunable luminescence of GOQDs and the antioxidant capacity of RGO [36,37]. Owing to the complexity of GO groups, GOQDs and RGO have become the most extensively explored GO derivatives. On that basis, more GO derivatives will be developed. GO and its derivatives can be prepared in conjunction with other materials through physical or chemical methods to form GO-based nanocomposites with excellent properties and broad prospects in biotechnology.

## 2. The Advantages of Using GO-Based Nanomaterials in the Treatment of Osteoporotic Fractures

GO can be combined with other materials to construct scaffolds, which can enhance the physical and chemical properties of these scaffolds, thereby promoting their efficacy in bone tissue repair. Furthermore, GO can be used to form coatings with other materials, which improves the mechanical properties, antimicrobial characteristics, and osteogenic capacity of bone implants, such as titanium alloys (Table 1). GO-based nanomaterials exhibit outstanding mechanical properties, favorable electrical conductivity, and exceptional biocompatibility. These attributes make them highly promising for application in the treatment of osteoporotic fractures.

### 2.1. Enhancing Material Properties to Promote Bone Tissue Repair: The Potential of GO and Its Derivatives

The mechanical properties of materials play a crucial role in providing structural support for osteoporotic fractures and significantly affect the regulation of cellular and tissue responses [52]. The composite of GO and its derivatives with other materials such as hydrogels can enhance the overall mechanical properties. For instance, the compression modulus of the Polylactic Acid/GO/Parathyroid Hormone composite scaffold, prepared by Fei et al., reached 2.64 MPa. This is nearly 110% higher compared with the compression modulus (1.26 MPa) of the scaffold without GO [53]. As confirmed in some studies, enhancing the electrical conductivity of materials can aid in bone tissue repair by inducing calcium influx to promote osteogenic differentiation and biomineralization [54]. Therefore, integrating materials with superior electrical conductivity is beneficial to bone tissue repair. GO, with its excellent electrical conductivity, can be composited with other materials to significantly enhance the osteogenic properties of the prepared material, thereby promoting the repair of osteoporotic fractures. Chen et al. utilized electrostatic spinning technology to prepare GO-based nanocomposites. The results demonstrate that the electrical conductivity of these GO-based nanocomposites improved as the proportion of GO increased, and the secretion of alkaline phosphatase (ALP) by bone marrow mesenchymal stem cells (BMSCs) in the GO group was significantly higher than that in the control group [42]. Calcium phosphate bone cement (CPC), derived from animal bone sintering, has been employed for the treatment of bone defects. However, their mechanical strength is relatively weak. To eliminate this defect, Seonwoo et al. prepared RGO-CPC nanomaterials. The mechanical properties of RGO-CPCs were significantly enhanced compared with those of CPCs, and both in vitro and in vivo experiments demonstrated that RGO-CPCs exhibited favorable biocompatibility and induced osteogenesis [43]. GO and its derivatives can improve the hydrophilicity and electrical conductivity of the material due to the abundance of oxygen-containing groups, which can promote cell adhesion and facilitate the proliferation and differentiation of cells. Baheti et al. deposited a hydroxyapatite/GO coating on titanium alloys. The results show that the hydrophilicity of this coating group was significantly higher than those of the uncoated group and the single hydroxyapatite coating group, and the adhesion and diffusion ability of BMSCs were enhanced [45]. Long et al. constructed RGO/Titanium dioxide (TiO_2_) nanocomposite coatings on the surface of titanium implants. The voltage-gated calcium channels were activated by the surface potential under the appropriate light intensity. The resulting light-induced surface potentials were harmless to the proliferative behavior of cells and facilitated the adsorption of the osteogenic growth factors. Further, these potentials exhibited osteogenic differentiation of BMSCs [49]. Tabatabaee et al. prepared scaffolds consisting of GO with gelatin and PHEMA. The compressive modulus, electrical conductivity, and hydrophilicity of these scaffolds increased with the addition of GO. The compressive modulus of these scaffolds increased significantly with the addition of GO at 0.75% *w*/*v* from 9.03 ± 0.36 MPa to 42.82 ± 1.63 MPa, while the conductivity of these scaffolds increased significantly from 4.48 ± 0.16 (* 10^−5^) S/m to 1.55 (* 10^−3^) S/m. The hydrophilicity of these scaffolds also increased significantly, which enhanced their osteoblastic ability [44]. However, osteoporosis was not modeled in the above study. Therefore, it is necessary to further explore the role of GO-based nanomaterials in osteoporosis animal models, as well as cellular models. In summary, the addition of GO and its derivatives can improve the mechanical, conductive, and hydrophilic properties of these materials, providing stable support as well as promoting the adhesion, diffusion, and osteogenic differentiation of BMSCs. Owing to their unique characteristics, GO-based nanocomposites are considered ideal implant materials for the treatment of osteoporotic fractures.

### 2.2. Harnessing Excellent Antimicrobial Properties: The Potential of GO-Based Nanomaterials

In clinical practice, the implantation of fixations in patients with fractures carries an inherent risk of infections [55]. Such infections often lead to delayed fracture healing or even non-union of fractures [56]. Most patients with osteoporotic fractures are the elderly. Their immune systems, which decline with age, are associated with disturbances in the immune microenvironment of bone tissues. This makes them more susceptible to postoperative infections [57,58]. When microorganisms form mature biofilms over time, the efficacy of antibiotics significantly decreases [59,60,61]. This situation can be largely mitigated by implanting fixations with antibacterial properties [62]. Due to their robust antimicrobial activity, GO-based nanocomposites can be employed to inhibit microbial adhesion by reducing the surface free energy (SFE) and preventing microbial adhesion through oxidative stress and photothermal (upon light activation at a specific wavelength, it converts to heat energy, causing local high temperature. This hampers microbial metabolism and denatures proteins/photodynamic effects (upon light activation at a specific wavelength, it generates oxygen free radicals and other free radical ions in the cell membrane. This triggers liposome peroxidation, disrupting the membrane’s integrity and causing content leakage, leading to microorganism inactivation) [63,64]. These mechanisms contribute to killing microorganisms and preventing the formation of microbial films. Guo et al. deposited GO coatings on the surface of sulfonated poly ether ether ketone (SPEEK) to form SPEEK-GO-based nanocomposites (SPEEK-GO). These composites demonstrated strong antimicrobial activity and promoted the adhesion, proliferation, and osteogenic differentiation of mouse embryonic osteoblast cells (MC3T3-E1) [50]. Han et al. prepared a berberine-loaded GO coating (Ber&GO@Ti) on biomedical titanium surfaces. The in vitro experiments showed that berberine exhibited low antimicrobial activity but enhanced antimicrobial activity against Staphylococcus aureus (*S. aureus*) due to the synergistic effect of GO and berberine. Furthermore, Ber&GO@Ti was biocompatible and promoted the osteogenic differentiation of MC3T3-E1. The in vivo experiments also showed excellent antibacterial properties and no infiltration of inflammatory cells in the surrounding tissues, such as neutrophils [48]. Fu et al. prepared gold nano-particles-poly(dopamine)-L-lysine functionalized-GO-PLGA composite scaffolds (AuNPs-PDA@PLGA/Lys-g-GO) for bone defect repair. These composite scaffolds displayed favorable mechanical strength, hydrophilicity, and antimicrobial properties. They significantly improved the in vitro adhesion, proliferation, and osteogenic differentiation of osteoblasts and significantly promoted new bone formation and collagen deposition at the radial defect site in vivo, demonstrating good biocompatibility [51]. The excellent mechanical properties, hydrophilicity, and electrical conductivity of GO-based nanocomposites can facilitate the rehabilitation in patients with osteoporotic fractures, while their robust antimicrobial activity can reduce the risk of infections during the rehabilitation process.

### 2.3. Promoting Bone Repair through Immune Regulation: The Potential of GO-Based Nanomaterials

Bone remodeling involves a process from bone resorption to bone formation, with these two phases being temporally and spatially coupled. This process takes place at the remodeling unit locus, where osteoclasts are initially recruited to resorb a significant amount of mineralized bone. Subsequently, osteoclasts undergo apoptosis, and osteoblasts are recruited to the site to form and mineralize new bone within the resorbed cavity [2]. In patients with osteoporosis, reduced estrogen levels, diabetes mellitus, prolonged use of glucocorticoids, rheumatoid arthritis, and other factors can hyperactivate osteoclast bone resorption while inhibiting osteoblast bone formation. This results in a disruption to bone metabolism balance, namely bone resorption exceeding bone formation, leading to bone loss and degradation of bone microstructures. Consequently, these patients become susceptible to osteoporotic fractures [65,66,67]. Therefore, balancing the functions of osteoclasts and osteoblasts is crucial in the treatment of osteoporotic fractures. Macrophages, precursor cells of osteoclasts, can participate in bone remodeling by secreting inflammatory factors such as tumor necrosis factor (TNF) α, interleukin (IL) 6, and IL-10. Hence, it is possible to indirectly balance the functions of osteoclasts and osteoblasts by regulating macrophages [68,69,70]. As revealed in most studies, macrophages are primarily classified into classic (M1) and atypical (M2) macrophages within the organismal microenvironment. M1-type macrophages mainly exhibit pro-inflammatory, antibacterial, and antigen-presenting functions, whereas M2-type macrophages primarily inhibit inflammation and facilitate tissue repair [71]. It has been demonstrated that GO-based nanocomposites can promote the polarization of M1 to M2 macrophages for regulating the immune microenvironment [72]. For instance, Fu et al. developed a GO-based composite hydrogel (Alg/GO/Ser/nHAP) that fostered conducive bone growth and bone immune microenvironment, which enhanced the osseointegration process at the bone–implant interface by shifting the macrophage phenotype from M1 to M2 [41]. Similarly, Xue et al. found that quaternized chitosan scaffolds with GO modification were more effective than pure quaternized chitosan scaffolds in promoting the polarization of M2-type macrophages and osteogenesis [73]. Shi et al. developed a GO composite hydrogel (PP/GO@PEGDA/CMC) which exhibited excellent mechanical properties, swelling capacity, and stability and significantly promoted M2-type polarization. This increased anti-inflammatory factors (IL-4, IL-10, and TGF-β), which in turn promoted the proliferation and osteogenic differentiation of BMSCs in vitro. The results further verify the anti-inflammatory effect of PP/GO@PEGDA/CMC in promoting bone regeneration in in vivo experiments [38]. Therefore, GO-based nanocomposites can regulate the immune microenvironment by promoting the polarization of M1 to M2-type macrophages and the secretion of anti-inflammatory factors, thereby promoting bone tissue repair. This presents a promising application prospect in the treatment of osteoporotic fractures.

### 2.4. Enhancing the Treatment of Osteoporotic Fractures: The Role of Drugs, Cells, and miRNA Loaded into GO-Based Nanomaterials

GO-based nanocomposites are promising carriers for drugs, stem cells, microRNAs, and other therapeutic agents (Table 2). These nanocomposites can be employed in the treatment of various diseases and play a significant role in tumor inhibition, angiogenesis, bacterial growth inhibition, and tissue repair [74,75,76]. Therapeutic agents, such as bisphosphonates, parathyroid hormone, strontium, BMSCs, and microRNAs, can be utilized to balance the functions of osteoclasts and osteoblasts, shifting from overactive bone resorption to normal bone metabolism, thus improving the microenvironment of osteoporotic fractures [77]. However, impaired blood circulation at the lesion site, instability of microRNAs in serum, and their intrinsic negative charge interfering with cellular uptake may induce insufficient drug concentrations and other challenges [78,79]. Additionally, BMSCs often struggle to efficiently undergo osteogenic differentiation due to the lack of a suitable microenvironment [77]. To overcome these challenges, GO-based nanocomposites can be used as carriers to achieve effective in situ delivery, thereby improving the therapeutic effect on osteoporotic fractures. For instance, Zeng et al. developed a controlled-release system based on a collagen-GO sponge loaded with alendronate sodium for the treatment of osteoporotic bone defects. This material prolonged the release of the drug, effectively inhibited the differentiation of monocyte-macrophages to osteoclasts, reduced bone loss in osteoporotic rats, and increased the volume of new bone at the defect site [80]. Similarly, Qin et al. prepared polyethylene glycol and polyethylene imide-functionalized GO nanocomplexes for the loading and delivery of miR-29b, which was involved in multiple steps of bone formation. The nanocomplexes presented favorable biocompatibility, microRNA loading capacity, and transfection efficiency, and the loading of miR-29b significantly promoted the osteogenic differentiation and bone regeneration of BMSCs [81]. Furthermore, Yu et al. developed a dual-channel GO composite scaffold encapsulating bone marrow-derived macrophages and BMSCs. In a rat cranial defect model, the scaffold effectively promoted the M2-type polarization of macrophages in the early bone defect microenvironment through the paracrine secretion of macrophages and BMSCs, thus avoiding excessive inflammatory responses and further promoting bone repair [82]. Neurons of the peripheral nervous system play a crucial role in regulating fracture healing by secreting neurotransmitters involved in bone growth and repair. However, osteoporotic fractures often struggle to achieve simultaneous nerve regeneration during the healing process, and persistent chronic pain is often associated with poor healing outcomes [83]. To remove this hindrance, Zhang et al. developed a GO-based hydrogel loaded with Schwann cells and BMSCs. The in vitro experiments demonstrated that the cells loaded on the hydrogel had high viability and good adhesion capacity. The in vivo experiments corroborated that the hydrogel could simultaneously promote the high expression of osteogenic and neural proteins, thus successfully promoting the synergistic regeneration of nerves and bones [84]. In summary, drugs, cells, microRNAs, and other therapeutic agents can be loaded onto GO-based nanomaterials to play an optimal role in treatment. This further enhances the effectiveness of GO-based nanocomposites in repairing osteoporotic fractures.

## 3. Potential Mechanisms and Related Signaling Pathways of Graphene Oxide-Based Materials in Promoting the Repair of Osteoporotic Fractures

**Figure 3:**
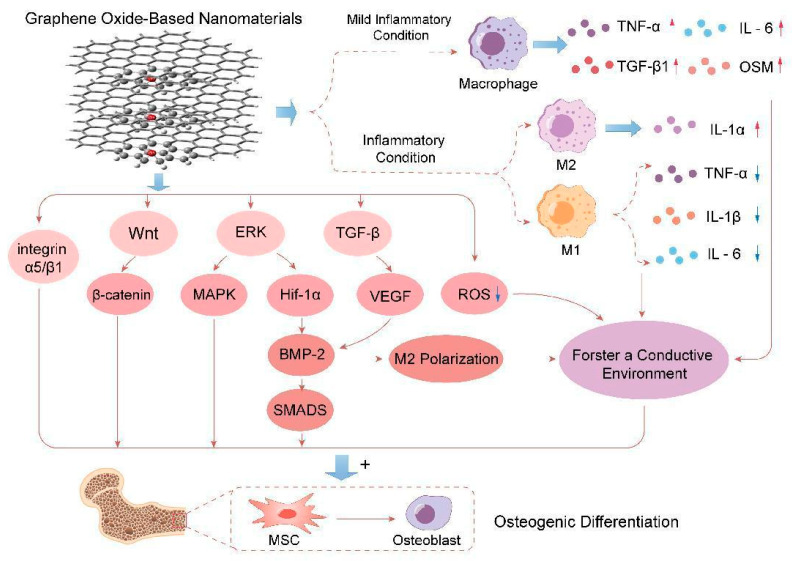
The mechanisms of GO-based nanomaterials affecting osteogenesis.

Due to the diversity of GO-based nanocomposites, the mechanism related to the repair of osteoporotic fractures may vary depending on the material (Figure 3). In the current study, the most important signaling pathways of GO-based nanocomposites to promote bone formation include the Wnt/β-catenin, BMPR/SMAD, and MAPK signaling pathways, and GO-based nanocomposites can regulate osteogenesis through the role of related pathways [86].

### 3.1. Common Signaling Pathways in the Promotion of Osteoporotic Fracture Repair by Graphene Oxide-Based Nanomaterials

Tissue repair is a multifaceted physiological process that necessitates the participation of various cell types, growth factors, cytokines, and signal transduction pathways [87]. As a classical pathway during bone formation, the Wnt/β-catenin signaling pathway is highly conserved throughout biological evolution and plays a pivotal role in bone regeneration. The enhancement of bone formation and regeneration by GO may be attributed to the activation of the Wnt/β-catenin pathway [88]. Xu et al. co-cultured BMSCs with different concentrations of GO derivatives and GO quantum dots (GOQDs). They found that GOQDs with a low concentration could promote the osteogenic differentiation of BMSCs through the activation of the Wnt/β-catenin signaling pathway [89]. Yang et al. discovered that GOQDs could also stimulate the proliferation and osteogenic differentiation of stem cells from human exfoliated deciduous teeth (SHED) via the Wnt/β-catenin pathway. They observed that the addition of the Wnt/β-catenin inhibitor DKK1 or the knockdown of β-catenin significantly down-regulated the expression of osteogenic-related mRNA and proteins [90]. The ERK/MAPK pathway, which is involved in various bone signaling responses, can up-regulate the expression of alkaline phosphatase (ALP), boost matrix mineralization, and promote osteogenic differentiation. In recent years, the MAPK signaling pathway has been identified as a key regulator of bone mass in osteogenic differentiation mediated by GO-based nanocomposites [86]. Zhao et al. unraveled that graphene composites (GNS-CaP-CS/AZ91D) could activate the ERK/MAPK signaling pathway to promote osteogenic differentiation through the sustained release of graphene nanosheets [91]. Chen et al. developed a strontium-GO-collagen scaffold (Sr-GO-Col) which significantly enhanced osteogenic regeneration and angiogenesis through the synergistic activation of the MAPK signaling pathway via GO and strontium [92]. Bone morphogenetic proteins (BMPs) are abundant in the bone matrix, with BMP-2 being the most crucial extracellular signaling molecule that promotes bone formation and induces osteogenic differentiation. Zhang et al. showed that graphene oxide-copper (GO-CU) nanocomposites activated the ERK1/2 signaling pathway. This led to the up-regulated expression of hypoxia-inducible factor 1-alpha (Hif-1α) in BMSCs, which ultimately resulted in the secretion of BMP-2, significantly inducing osteogenic differentiation [93]. Yi He et al. prepared a magnetic GO composed of ferric iron tetroxide (Fe_3_O_4_) and GO. This material significantly accelerated the osteogenic differentiation of BMSCs by activating the BMP signaling pathway and promoting the expression of BMP-2. These experimental results suggested that GO can either promote osteogenesis through specific signaling pathways or synergistically stimulate relevant signaling pathways with other materials to accelerate bone tissue regeneration and resolve osteoporotic fractures. There are extensive and complex networks of mechanisms by which GO-based nanocomposites regulate osteogenic differentiation, with various pathways interconnecting and interacting with each other to promote osteogenic differentiation. Shim et al. found that polydopamine-graphene oxide (PDA/GO) composites stimulated the osteogenic differentiation of mouse embryonic stem cells through integrin α5/β1, MAPK, and BMPR/SMAD signaling pathways. The levels of integrins α5 and β1, as well as bone morphogenetic protein receptor (BMPR) type I and type II, were significantly elevated in mouse embryonic stem cells on PDA/GO composites. The expression of BMPs phosphorylated by SMAD1/5/8 was significantly up-regulated, and the phosphorylation of ERKs and MAPKs was also observed. After blocking integrins α5/β1, MAPK, or SMAD signaling pathways, the osteogenic differentiation of embryonic stem cells induced by PDA/GO was significantly reduced [94].

### 3.2. Mechanisms of Bone Tissue Repair Promoted by Graphene Oxide Composite-Based Nanomaterials through Immune Regulation

Disorders of the immune system are recognized as one of the pathogenic mechanisms underlying osteoporosis [95]. Upon implantation, bone biomaterials are identified by the immune system, triggering corresponding immune responses that can influence the efficacy of bone repair [96]. Consequently, the immunomodulatory function of bone biomaterials warrants emphasis [97]. As a category of bone biomaterials with immunomodulatory properties, GO-based nanocomposites are of particular interest. Investigating their immunomodulatory mechanisms may provide clues for the development of bone biomaterials with similar properties [98,99]. Su et al. confirmed that GO coatings could modulate macrophage polarization and cytokine secretion via Toll-like receptors. Under normal conditions, GO coatings induced a mild inflammatory response and fostered a conducive environment for bone formation by stimulating macrophages to secrete minimal amounts of inflammatory factors (TNF-α and IL-6) and osteogenic factors (TGF-β1 and OSM). In contrast, under inflammatory conditions, GO coatings down-regulated the expression of inflammatory factors in M1-type macrophages by inhibiting the excessive secretion of inflammatory factors (TNF-α, IL-6, and IL-1β) and up-regulated the expression of IL-1ra in M2-type macrophages, thereby mitigating the inflammatory response [100]. Zhou et al. utilized a reduced graphene oxide hydrogel (GM/Ac-CD/rGO) in a mouse cranial defect model. They found that the hydrogel could enhance the immune microenvironment by neutralizing free oxygen radicals (ROS) around the cranial defect through the electron transfer capacity of reduced GO [101]. In a study by Hang Xue et al., it was demonstrated that quaternized chitosan-graphene oxide-polydopamine nanocomposites (QCS-GO-PDA) significantly scavenged ROS and reduced inflammatory responses. This was achieved by activating TGF-β/BMP2, VEGF, and other signaling pathways to promote the polarization of M2-type macrophages and augment the immune crosstalk between bone and angiogenesis [73]. Therefore, GO-based nanocomposites may enhance the bone immune microenvironment by neutralizing oxygen radicals produced by inflammation. Simultaneously, they may regulate the polarization of macrophages by activating specific signaling pathways, such as TGF-β/BMP2 and VEGF. These properties could potentially facilitate the repair of osteoporotic fractures.

## 4. Cytotoxicity of Graphene Oxide Limits Its Application in Tissue Engineering

In recent years, there has been increasing research on GO and its use in tissue engineering. However, concerns about the safety of GO-based nanomaterials have also been raised. Some studies have shown that GO itself has cytotoxic effects. It has been confirmed that GO can induce immune responses and toxicity in adult zebrafish. Researchers have suggested that GO can mediate apoptosis through the ROS/AMPK/p53 signaling pathway, leading to inflammation and inflammatory diseases [102,103]. Despite efforts to improve the purity of GO during synthesis, there is still some cytotoxicity associated with impurities. This suggests that cytotoxicity is an inherent property of GO [104]. In vivo, GO degrades and releases nanoparticles, which can cause cytotoxicity. The morphology of GO, its chemical composition, the timing and dosage of its release, and the biological environment all play important roles in its cytotoxic effects [105]. GO and its derivatives have been found to down-regulate the expression of genes related to the cell membrane and cytoskeleton, leading to disruption of cell membrane integrity and loss of normal cellular metabolism [106]. Studies have shown that exposure to GO materials can cause mitochondrial and plasma membrane damage in HaCaT cells, and the extent of graphene oxidation can exacerbate cellular damage [107]. In addition, nanosheets released from high concentrations of GO can perforate the cell membrane and accelerate cell death in A549 and Raw264.7 cells in vitro [108]. The cytotoxicity of GO limits its potential applications in tissue engineering. However, efforts to improve the biocompatibility of GO or modify its molecule to regulate the release of nanoparticles may help reduce its cytotoxic effects [109]. Currently, the mitigation of GO cytotoxicity in vivo is still limited, and further research is urgently needed to advance its applications in vivo.

## 5. Conclusions and Perspective

Osteoporotic fractures pose a significant treatment challenge due to the individual characteristics of patients, the properties of implant materials, and the nature of the disease itself. Both domestic and international studies have confirmed that GO-based nanomaterials possess excellent mechanical properties, favorable biocompatibility, and the ability to induce osteogenesis. These advantages make them suitable for their application in the treatment of osteoporotic fractures.

As mentioned earlier, GO-based nanocomposites have shown excellent potential for promoting bone formation and inhibiting bone resorption. GO composite scaffolds and coatings are commonly used for the treatment of osteoporotic fractures. These composites address the limitations of single-material scaffolds, such as poor mechanical properties, low electrical conductivity, and low antimicrobial capacity. Compared to endoprostheses like titanium alloys, GO composite coatings offer advantages like induced osteogenesis and improved antimicrobial capacity, resulting in better therapeutic effects for osteoporotic fractures.

To enhance the osteogenic effect of GO composites, researchers have developed various engineering strategies, including drug delivery and surface modification. However, these strategies may also have drawbacks, such as uncertain immune responses and high production costs. GO-based nanocomposites may be recognized by the immune system as foreign substances, triggering an immune response that could reduce their efficacy or lead to clearance. Additionally, GO-based nanocomposites may cause toxicity or adverse reactions, posing potential risks to the host. The development and research of GO composites require additional time, expenses, and technology, which can increase production costs. Therefore, it is crucial to consider safety, immunogenicity, stability, and production costs when designing GO composites. Rigorous experimental and clinical studies are necessary to evaluate their application prospects. Despite the promising potential of GO composites in osteoporosis management, there are still challenges that need to be addressed. Methods for preparing and testing the safety of GO composites are not fully developed. It is important to understand how their composition and preparation processes affect their bioactivity and stability. Furthermore, studying the function and regulatory mechanisms of bioactive substances in GO complexes is necessary to determine how they can effectively treat osteoporosis.

Future research should focus on exploring more efficient and stable methods for the preparation of GO-based nanomaterials. In-depth studies on their biological mechanisms are needed, and clinical trials should be conducted to promote the application of GO nanocomposites in osteoporosis treatment.

## Figures and Tables

**Table 1:** Use of GO-based nanomaterials for bone tissue repair.

Type	Name	Compose	Animal Species	Cytotoxicity Tests	Biocompatibility	Conclusion	Reference
Scaffold	PP/GO@PEGDA/CMC	GO, Pyritum, Polydiacrylate, Carboxymethyl chitosan	SD rats with bilateral skull defect model	Non-toxic to macrophages	Induced macrophage polarizes to M2 phenotype	It has both immunomodulatory and osteoinductive properties	[38]
	PLA-HT-GO	Polylactic acid, Hardystonite, GO	NA	Non-toxic to MG63	Enhanced the proliferation and adhesion of MG63	HT-GO nanoparticles improved the mechanical properties and osteoinductive properties	[39]
	Z-CS/β-TCP/GO	GO, Maleicanhydride, L-cysteine, Maleilated chitosan	SD rats with bilateral skull defect model	Non-toxic to BMSCs	Enhanced the proliferation and adhesion of BMSCs	GO improved physicochemical properties and osteogenic differentiation of rBMSCs	[40]
	Alg/GO/Ser/nHAP	Alginate, GO, Sericin, Nanohydroxyapatite	SD rats with bilateral skull defect	Non-toxic to BMSCs	1. Induced macrophage polarizes to M2 phenotype 2. Enhanced the proliferation and adhesion of BMSCS	It has both immunomodulatory and osteoinductive properties	[41]
	GO-PVDF	GO, Polyvinylidene fluoride	NA	Non-toxic to osteoprogenitor D1 cells	Enhanced the proliferation and mineralization of osteoprogenitor D1 cells	Exhibits excellent tensile and piezoelectric properties, high flexibility, and good biocompatibility	[42]
	RGO-CPCs	RGO, Calcium phosphate cements	SD rats with bilateral skull defect model	Slight toxicity at 12 h and became marginal at 24 h to MC3T3-E1 and rASCs	Improved osteogenic differentiation of MC3T3-E1 and rASCs	The mechanical properties and the bone-inducing activity of the rGO-CPCs were enhanced in comparison with CPCs	[43]
	PHEMA-Gel-GO	Poly(2-hydroxyethyl methacrylate), Geltain, GO	NA	Non-toxic to BMSCs	Enhanced the proliferation and adhesion of BMSCs	Mechanical properties, electroactivity, and osteogenic differentiation ability can be improved through the GO	[44]
Coating	HA-GO-Ti	Hydroxyapatite, GO, Ti	SD rats with distal femurs defect model	Non-toxic to BMSCs	Enhanced the proliferation and adhesion of BMSCs	HA-GO nanocoating improve the osteogenesis of the Ti	[45]
	Mg(OH)2/GO/HA-ZQ71	Mg(OH)2, GO, Hydroxyapatite, ZQ71 alloy	NA	Non-toxic to MC3T3-E1	Enhanced the proliferation and adhesion of MC3T3-E1	Mg(OH)2/GO/HA composite coating improved the corrosion resistance and osteogenesis and reduced the degradation rate	[46]
	Ag-RGO-Ti	Ag, rGO, Ti	NA	Non-toxic to MC3T3-E1	Enhanced the proliferation and adhesion of MC3T3-E1	RGO+Ag coating improved the antibacterial activity and osteogenesis of Ti	[47]
	Berberine-GO-Ti	Berberine, GO, Ti	SD rats with distal femurs defect model	Non-toxic to MC3T3-E1	Enhanced the proliferation and adhesion of MC3T3-E1	Berberine-loaded GO coating improved the antibacterial activity and osteogenesis of Ti	[48]
	GO/TiO_2_/Ti	GO, TiO_2_, Ti	NA	Non-toxic to BMSCs	Enhanced the adhesion of BMSCs	The rGO/TiO_2_ has good biocompatibility and light-induced surface potential and could promote BMSC osteogenic differentiation	[49]
	SPEEK-GO	GO, Poly-ether-ether-ketone, 98% sulfuric acid	NA	Non-toxic to MC3T3-E1	Enhanced the proliferation and adhesion of MC3T3-E1	The SPEEK-GO coating exhibits strong antibacterial properties and excellent cell compatibility	[50]
	AuNPs-PDA@PLGA/Lys-g-GO	L-lysine functionalized-GO, Polydopamine, Au, PLGA	Rabbits with radius defects	Non-toxic to MC3T3-E1	Enhanced the proliferation and adhesion of MC3T3-E1	Lys-g-GO nanoparticles and AuNPs-PDA coating enhanced the hydrophilicity, mechanical properties, and antibacterial properties, resulting in good osteogenic activity.	[51]

**Table 2:** GO-based nanomaterials as carriers for drugs/cells/miRNA in bone tissue repair therapy.

Name	Compose	Animal Species	Cytotoxicity Tests	Biocompatibility	Conclusion	Reference
PCL/GO-DEX	Polycaprolactone, GO, Dexamethasone	NA	Non-toxic to BMSCs	Enhanced the proliferation and adhesion of BMSCs	The synergistic effect of GO and dexamethasone induce osteogenesis of BMSCs	[85]
Aln-GO-Col	Alendronate, Collagen, GO	SDrats with bilateral skull defect model	Toxic to BMSCs at high concentration of GO	1. Enhanced the proliferation and adhesion of BMSCs 2. Inhibited osteoclast differentiation	Exhibited active anti-osteoclastogenesis and osteogenesis ability	[80]
PLA/GO/RhPTH(1-34)	Polylactide, GO, RhPTH(1-34)	NA	Non-toxic to MC3T3-E1	Enhanced the proliferation and adhesion of MC3T3-E1	Mechanical properties and osteogenic differentiation ability can be improved through the scaffolds	[53]
miR-29b/GO-PEG-PEI@CS	GO, Polyethyleneglycol, Polyethylenimine, miR-29b, Chitosan	SD rats with bilateral skull defect model	Non-toxic to BMSCs	Enhanced the proliferation and adhesion of BMSCs	It can promote bone regeneration without inflammatory responses	[81]
BMSCS/SCS/rGO/GelMA	BMSCs, SCs, rGO, GelMA	SD rats with bilateral skull defect model	Non-toxic to BMSCs and SCs	Enhanced the proliferation and adhesion of BMSCs and SCs	It can promote synergistic regeneration of nerves and bone	[84]
BMSCS/BMMS/GelMA/HAMA/Alginate/GO	BMSCS, BMMS, Methacrylamidated gelatin, Methacrylamidated Hyaluronic, Alginate	Rat subcutaneous transplantation model	Non-toxic to BMSCs	1.Induced macrophage polarizes to M2 phenotype 2.Enhanced the proliferation and adhesion of BMSCs	The paracrine of BMMs and BMSCs effectively promoted the M2-type polarization and bone repair.	[82]

## References

[1] (1993). Consensus development conference: Diagnosis, prophylaxis, and treatment of osteoporosis. Am. J. Med..

[2] Compston J.E., McClung M.R., Leslie W.D. (2019). Osteoporosis. Lancet.

[3] Gruber R., Koch H., Doll B.A., Tegtmeier F., Einhorn T.A., Hollinger J.O. (2006). Fracture healing in the elderly patient. Exp. Gerontol..

[4] Kammerlander C., Gebhard F., Meier C., Lenich A., Linhart W., Clasbrummel B., Neubauer-Gartzke T., Garcia-Alonso M., Pavelka T., Blauth M. (2011). Standardised cement augmentation of the PFNA using a perforated blade: A new technique and preliminary clinical results. A prospective multicentre trial. Injury.

[5] Hollensteiner M., Sandriesser S., Bliven E., von Rüden C., Augat P. (2019). Biomechanics of Osteoporotic Fracture Fixation. Curr. Osteoporos. Rep..

[6] Osterhoff G., Morgan E.F., Shefelbine S.J., Karim L., McNamara L.M., Augat P. (2016). Bone mechanical properties and changes with osteoporosis. Injury.

[7] Maki B.E., McIlroy W.E. (2006). Control of rapid limb movements for balance recovery: Age-related changes and implications for fall prevention. Age Ageing.

[8] Gale C.R., Cooper C., Aihie Sayer A. (2016). Prevalence and risk factors for falls in older men and women: The English Longitudinal Study of Ageing. Age Ageing.

[9] Röderer G., Scola A., Schmölz W., Gebhard F., Windolf M., Hofmann-Fliri L. (2013). Biomechanical in vitro assessment of screw augmentation in locked plating of proximal humerus fractures. Injury.

[10] Wu X., Wang Z., Li H., Li Y., Wang H., Tian W. (2019). Biomechanical evaluation of osteoporotic fracture: Metal fixation versus absorbable fixation in Sawbones models. Injury.

[11] von Rüden C., Augat P. (2016). Failure of fracture fixation in osteoporotic bone. Injury.

[12] Chen X.J., Zhang X.Q., Liu Q., Zhang J., Zhou G. (2018). Nanotechnology: A promising method for oral cancer detection and diagnosis. J. Nanobiotechnol..

[13] Ziąbka M., Menaszek E., Tarasiuk J., Wroński S. (2018). Biocompatible Nanocomposite Implant with Silver Nanoparticles for Otology-In Vivo Evaluation. Nanomaterials.

[14] Cheng X., Wan Q., Pei X. (2018). Graphene Family Materials in Bone Tissue Regeneration: Perspectives and Challenges. Nanoscale Res. Lett..

[15] Mahmoudi E., Ang W.L., Ng C.Y., Ng L.Y., Mohammad A.W., Benamor A. (2019). Distinguishing characteristics and usability of graphene oxide based on different sources of graphite feedstock. J. Colloid. Interf. Sci..

[16] Xie H., Cao T., Rodríguez-Lozano F.J., Luong-Van E.K., Rosa V. (2017). Graphene for the development of the next-generation of biocomposites for dental and medical applications. Dent. Mater..

[17] Govindarajan D., Saravanan S., Sudhakar S., Vimalraj S. (2023). Graphene: A Multifaceted Carbon-Based Material for Bone Tissue Engineering Applications. ACS Omega.

[18] Sofer Z., Jankovský O., Šimek P., Sedmidubský D., Šturala J., Kosina J., Mikšová R., Macková A., Mikulics M., Pumera M. (2015). Insight into the mechanism of the thermal reduction of graphite oxide: Deuterium-labeled graphite oxide is the key. ACS Nano.

[19] Miao Y., Wang X., Liu Y., Liu Z., Chen W. (2021). Preparation of Graphene Oxide/Cellulose Composites with Microcrystalline Cellulose Acid Hydrolysis Using the Waste Acids Generated by the Hummers Method of Graphene Oxide Synthesis. Polymers.

[20] Soltani T., Lee B.-K. (2017). Low intensity-ultrasonic irradiation for highly efficient, eco-friendly and fast synthesis of graphene oxide. Ultrason. Sonochem.

[21] Yu H., Zhang B., Bulin C., Li R., Xing R. (2016). High-efficient Synthesis of Graphene Oxide Based on Improved Hummers Method. Sci. Rep..

[22] Wang Y., Li Z., Wang J., Li J., Lin Y. (2011). Graphene and graphene oxide: Biofunctionalization and applications in biotechnology. Trends Biotechnol..

[23] Kinloch I.A., Suhr J., Lou J., Young R.J., Ajayan P.M. (2018). Composites with carbon nanotubes and graphene: An outlook. Science.

[24] Yuan Z., Xiao X., Li J., Zhao Z., Yu D., Li Q. (2018). Self-Assembled Graphene-Based Architectures and Their Applications. Adv. Sci..

[25] Nanda S.S., Yi D.K., Kim K. (2016). Study of antibacterial mechanism of graphene oxide using Raman spectroscopy. Sci. Rep..

[26] Li Y., Zhao Y., Cheng H., Hu Y., Shi G., Dai L., Qu L. (2012). Nitrogen-doped graphene quantum dots with oxygen-rich functional groups. J. Am. Chem. Soc..

[27] Lim S.Y., Shen W., Gao Z. (2015). Carbon quantum dots and their applications. Chem. Soc. Rev..

[28] Lin L., Zhang S. (2012). Creating high yield water soluble luminescent graphene quantum dots via exfoliating and disintegrating carbon nanotubes and graphite flakes. Chem. Commun..

[29] Lin T.N., Chih K.H., Yuan C.T., Shen J.L., Lin C.a.J., Liu W.R. (2015). Laser-ablation production of graphene oxide nanostructures: From ribbons to quantum dots. Nanoscale.

[30] Huang Z., Shen Y., Li Y., Zheng W., Xue Y., Qin C., Zhang B., Hao J., Feng W. (2014). Facile synthesis of analogous graphene quantum dots with sp(2) hybridized carbon atom dominant structures and their photovoltaic application. Nanoscale.

[31] Li X., Cai W., An J., Kim S., Nah J., Yang D., Piner R., Velamakanni A., Jung I., Tutuc E. (2009). Large-area synthesis of high-quality and uniform graphene films on copper foils. Science.

[32] Foroutan T., Nazemi N., Tavana M., Kassaee M.Z., Motamedi E., Soieshargh S., Zare Zardini H. (2018). Suspended graphene oxide nanoparticle for accelerated multilayer osteoblast attachment. J. Biomed. Mater. Res. A.

[33] Sokolov D.A., Shepperd K.R., Orlando T.M. (2010). Formation of Graphene Features from Direct Laser-Induced Reduction of Graphite Oxide. J. Phys. Chem. Lett..

[34] Rai V.K., Mahata S., Kashyap H., Singh M., Rai A. (2020). Bio-reduction of Graphene Oxide: Catalytic Applications of (Reduced) GO in Organic Synthesis. Curr. Org. Synth..

[35] Gilje S., Dubin S., Badakhshan A., Farrar J., Danczyk S.A., Kaner R.B. (2010). Photothermal deoxygenation of graphene oxide for patterning and distributed ignition applications. Adv. Mater..

[36] Tan Y., Chen Y., Lu T., Witman N., Yan B., Gong Y., Ai X., Yang L., Liu M., Luo R. (2023). Engineering a conduction-consistent cardiac patch with rGO/PLCL electrospun nanofibrous membranes and human iPSC-derived cardiomyocytes. Front. Bioeng. Biotech..

[37] Ghasemlou M., Mayes E.L.H., Murdoch B.J., Le P.H., Dekiwadia C., Aburto-Medina A., Daver F., Ivanova E.P., Adhikari B. (2022). Silicon-Doped Graphene Oxide Quantum Dots as Efficient Nanoconjugates for Multifunctional Nanocomposites. ACS Appl. Mater. Interfaces.

[38] Shi C., Yu Y., Wu H., Liu H., Guo M., Wang W., Wang D., Wei C., Zhai H., Yan G. (2023). A graphene oxide-loaded processed pyritum composite hydrogel for accelerated bone regeneration via mediation of M2 macrophage polarization. Mater. Today Bio.

[39] Tavakoli M., Emadi R., Salehi H., Labbaf S., Varshosaz J. (2023). Incorporation of graphene oxide as a coupling agent in a 3D printed polylactic acid/hardystonite nanocomposite scaffold for bone tissue regeneration applications. Int. J. Biol. Macromol..

[40] Wang Q., Li M., Cui T., Wu R., Guo F., Fu M., Zhu Y., Yang C., Chen B., Sun G. (2023). A Novel Zwitterionic Hydrogel Incorporated with Graphene Oxide for Bone Tissue Engineering: Synthesis, Characterization, and Promotion of Osteogenic Differentiation of Bone Mesenchymal Stem Cells. Int. J. Mol. Sci..

[41] Fu M., Li J., Liu M., Yang C., Wang Q., Wang H., Chen B., Fu Q., Sun G. (2023). Sericin/Nano-Hydroxyapatite Hydrogels Based on Graphene Oxide for Effective Bone Regeneration via Immunomodulation and Osteoinduction. Int. J. Nanomed..

[42] Chen W.-C., Huang B.-Y., Huang S.-M., Liu S.-M., Chang K.-C., Ko C.-L., Lin C.-L. (2023). In vitro evaluation of electrospun polyvinylidene fluoride hybrid nanoparticles as direct piezoelectric membranes for guided bone regeneration. Mater. Sci. Eng. C.

[43] Seonwoo H., Choung H.-W., Park S., Choi K.S., Jang K.-J., Kim J., Lim K.-T., Kim Y., Garg P., Pandey S. (2022). Reduced graphene oxide-incorporated calcium phosphate cements with pulsed electromagnetic fields for bone regeneration. RSC Adv..

[44] Tabatabaee S., Baheiraei N., Salehnia M. (2022). Fabrication and characterization of PHEMA-gelatin scaffold enriched with graphene oxide for bone tissue engineering. J. Orthop. Surg. Res..

[45] Baheti W., Lv S., Mila, Ma L., Amantai D., Sun H., He H. (2023). Graphene/hydroxyapatite coating deposit on titanium alloys for implant application. J. Appl. Biomater. Func..

[46] Yuan B., Chen H., Zhao R., Deng X., Chen G., Yang X., Xiao Z., Aurora A., Iulia B.A., Zhang K. (2022). Construction of a magnesium hydroxide/graphene oxide/hydroxyapatite composite coating on Mg-Ca-Zn-Ag alloy to inhibit bacterial infection and promote bone regeneration. Bioact. Mater..

[47] San H., Paresoglou M., Minneboo M., van Hengel I.A.J., Yilmaz A., Gonzalez-Garcia Y., Fluit A.C., Hagedoorn P.-L., Fratila-Apachitei L.E., Apachitei I. (2022). Fighting Antibiotic-Resistant Bacterial Infections by Surface Biofunctionalization of 3D-Printed Porous Titanium Implants with Reduced Graphene Oxide and Silver Nanoparticles. Int. J. Mol. Sci..

[48] Han X.-Y., Meng T., Ye J.-X., Yin H.-B., Song D.-W. (2022). Enhanced Antibacterial and Osteogenic Properties of Graphene Oxide Loaded with Berberine on Biomedical Titanium. J. Biomed. Nanotechnol..

[49] Long X., Duan L., Weng W., Cheng K., Wang D., Ouyang H. (2021). Light-induced osteogenic differentiation of BMSCs with graphene/TiO2 composite coating on Ti implant. Colloids Surf. B Biointerfaces.

[50] Guo C., Lu R., Wang X., Chen S. (2021). Antibacterial activity, bio-compatibility and osteogenic differentiation of graphene oxide coating on 3D-network poly-ether-ether-ketone for orthopaedic implants. J. Mater. Sci. Mater. Med..

[51] Fu C., Jiang Y., Yang X., Wang Y., Ji W., Jia G. (2021). Mussel-Inspired Gold Nanoparticle and PLGA/L-Lysine-g-Graphene Oxide Composite Scaffolds for Bone Defect Repair. Int. J. Nanomed..

[52] He Y., Zhao Y., Fan L., Wang X., Duan M., Wang H., Zhu X., Liu J. (2021). Injectable Affinity and Remote Magnetothermal Effects of Bi-Based Alloy for Long-Term Bone Defect Repair and Analgesia. Adv. Sci..

[53] Fei F., Yao H., Wang Y., Wei J. (2023). Graphene Oxide/RhPTH(1-34)/Polylactide Composite Nanofibrous Scaffold for Bone Tissue Engineering. Int. J. Mol. Sci..

[54] Huang Y., Deng H., Fan Y., Zheng L., Che J., Li X., Aifantis K.E. (2019). Conductive nanostructured Si biomaterials enhance osteogeneration through electrical stimulation. Mater. Sci. Eng. C.

[55] Shen J., Sun D., Fu J., Wang S., Wang X., Xie Z. (2021). Management of surgical site infection post-open reduction and internal fixation for tibial plateau fractures. Bone Jt. Res..

[56] Medda S., Hsu J.R. (2022). Simplified Antibiotic-Coated Plating for Infected Nonunion, Fracture-Related Infection, and Single-Stage Prophylactic Fixation. J. Orthop. Trauma..

[57] Li J., Wong R.M.Y., Chung Y.L., Leung S.S.Y., Chow S.K.-H., Ip M., Cheung W.-H. (2022). Fracture-related infection in osteoporotic bone causes more severe infection and further delays healing. Bone Jt. Res..

[58] Zhang X., Man K.-W., Li G.H.-Y., Tan K.C., Kung A.W.-C., Cheung C.-L. (2022). Osteoporosis is a novel risk factor of infections and sepsis: A cohort study. EClinicalMedicine.

[59] Tomizawa T., Nishitani K., Ito H., Okae Y., Morita Y., Doi K., Saito M., Ishie S., Yoshida S., Murata K. (2021). The limitations of mono- and combination antibiotic therapies on immature biofilms in a murine model of implant-associated osteomyelitis. J. Orthop. Res..

[60] Masters E.A., Ricciardi B.F., Bentley K.L.d.M., Moriarty T.F., Schwarz E.M., Muthukrishnan G. (2022). Skeletal infections: Microbial pathogenesis, immunity and clinical management. Nat. Rev. Microbiol..

[61] Davies D. (2003). Understanding biofilm resistance to antibacterial agents. Nat. Rev. Drug Discov..

[62] Keller D.M., Pizzo R.A., Patel J.N., Viola A., Yoon R.S., Liporace F.A. (2022). Use of antibiotic-cement coated locking plates in the setting of periprosthetic infection and infected nonunion. Injury.

[63] Zou X., Zhang L., Wang Z., Luo Y. (2016). Mechanisms of the Antimicrobial Activities of Graphene Materials. J. Am. Chem. Soc..

[64] Agarwalla S.V., Ellepola K., Silikas N., Castro Neto A.H., Seneviratne C.J., Rosa V. (2021). Persistent inhibition of Candida albicans biofilm and hyphae growth on titanium by graphene nanocoating. Dent. Mater..

[65] Song S., Guo Y., Yang Y., Fu D. (2022). Advances in pathogenesis and therapeutic strategies for osteoporosis. Pharmacol. Therapeut.

[66] Black D.M., Rosen C.J. (2016). Clinical Practice. Postmenopausal Osteoporosis. N. Engl. J. Med..

[67] Buckley L., Humphrey M.B. (2018). Glucocorticoid-Induced Osteoporosis. N. Engl. J. Med..

[68] McDonald M.M., Khoo W.H., Ng P.Y., Xiao Y., Zamerli J., Thatcher P., Kyaw W., Pathmanandavel K., Grootveld A.K., Moran I. (2021). Osteoclasts recycle via osteomorphs during RANKL-stimulated bone resorption. Cell.

[69] Kim T.-H., Yang K., Kim M., Kim H.-S., Kang J.L. (2021). Apoptosis inhibitor of macrophage (AIM) contributes to IL-10-induced anti-inflammatory response through inhibition of inflammasome activation. Cell Death Dis..

[70] Sinder B.P., Pettit A.R., McCauley L.K. (2015). Macrophages: Their Emerging Roles in Bone. J. Bone Miner. Res..

[71] Schlundt C., Fischer H., Bucher C.H., Rendenbach C., Duda G.N., Schmidt-Bleek K. (2021). The multifaceted roles of macrophages in bone regeneration: A story of polarization, activation and time. Acta Biomater..

[72] Xue D., Chen E., Zhong H., Zhang W., Wang S., Joomun M.U., Yao T., Tan Y., Lin S., Zheng Q. (2018). Immunomodulatory properties of graphene oxide for osteogenesis and angiogenesis. Int. J. Nanomed..

[73] Xue H., Zhang Z., Lin Z., Su J., Panayi A.C., Xiong Y., Hu L., Hu Y., Chen L., Yan C. (2022). Enhanced tissue regeneration through immunomodulation of angiogenesis and osteogenesis with a multifaceted nanohybrid modified bioactive scaffold. Bioact. Mater..

[74] Karimi Shervedani R., Mirhosseini H., Samiei Foroushani M., Torabi M., Rahsepar F.R., Norouzi-Barough L. (2018). Immobilization of methotrexate anticancer drug onto the graphene surface and interaction with calf thymus DNA and 4T1 cancer cells. Bioelectrochemistry.

[75] D’Amora U., Dacrory S., Hasanin M.S., Longo A., Soriente A., Kamel S., Raucci M.G., Ambrosio L., Scialla S. (2023). Advances in the Physico-Chemical, Antimicrobial and Angiogenic Properties of Graphene-Oxide/Cellulose Nanocomposites for Wound Healing. Pharmaceutics.

[76] Wu X., Li H., Xiao N. (2018). Advancement of Near-infrared (NIR) laser interceded surface enactment of proline functionalized graphene oxide with silver nanoparticles for proficient antibacterial, antifungal and wound recuperating therapy in nursing care in hospitals. J. Photochem. Photobiol. B.

[77] Cao L., Liu G., Gan Y., Fan Q., Yang F., Zhang X., Tang T., Dai K. (2012). The use of autologous enriched bone marrow MSCs to enhance osteoporotic bone defect repair in long-term estrogen deficient goats. Biomaterials.

[78] Roche B., Vanden-Bossche A., Malaval L., Normand M., Jannot M., Chaux R., Vico L., Lafage-Proust M.-H. (2014). Parathyroid hormone 1-84 targets bone vascular structure and perfusion in mice: Impacts of its administration regimen and of ovariectomy. J. Bone Miner. Res..

[79] Bai Z., Wei J., Yu C., Han X., Qin X., Zhang C., Liao W., Li L., Huang W. (2019). Non-viral nanocarriers for intracellular delivery of microRNA therapeutics. J. Mater. Chem. B.

[80] Zeng Y., Zhou M., Chen L., Fang H., Liu S., Zhou C., Sun J., Wang Z. (2020). Alendronate loaded graphene oxide functionalized collagen sponge for the dual effects of osteogenesis and anti-osteoclastogenesis in osteoporotic rats. Bioact. Mater..

[81] Qin H., Ji Y., Li G., Xu X., Zhang C., Zhong W., Xu S., Yin Y., Song J. (2022). MicroRNA-29b/graphene oxide-polyethyleneglycol-polyethylenimine complex incorporated within chitosan hydrogel promotes osteogenesis. Front. Chem..

[82] Yu K., Huangfu H., Qin Q., Zhang Y., Gu X., Liu X., Zhang Y., Zhou Y. (2022). Application of Bone Marrow-Derived Macrophages Combined with Bone Mesenchymal Stem Cells in Dual-Channel Three-Dimensional Bioprinting Scaffolds for Early Immune Regulation and Osteogenic Induction in Rat Calvarial Defects. ACS Appl. Mater. Interfaces.

[83] Wank I., Niedermair T., Kronenberg D., Stange R., Brochhausen C., Hess A., Grässel S. (2022). Influence of the Peripheral Nervous System on Murine Osteoporotic Fracture Healing and Fracture-Induced Hyperalgesia. Int. J. Mol. Sci..

[84] Zhang X., Zhang H., Zhang Y., Huangfu H., Yang Y., Qin Q., Zhang Y., Zhou Y. (2023). 3D printed reduced graphene oxide-GelMA hybrid hydrogel scaffolds for potential neuralized bone regeneration. J. Mater. Chem. B.

[85] Rostami F., Tamjid E., Behmanesh M. (2020). Drug-eluting PCL/graphene oxide nanocomposite scaffolds for enhanced osteogenic differentiation of mesenchymal stem cells. Mater. Sci. Eng. C Mater. Biol. Appl..

[86] Wu M., Zou L., Jiang L., Zhao Z., Liu J. (2021). Osteoinductive and antimicrobial mechanisms of graphene-based materials for enhancing bone tissue engineering. J. Tissue Eng. Regen. M..

[87] Chen Z., Klein T., Murray R.Z., Crawford R., Chang J., Wu C., Xiao Y. (2016). Osteoimmunomodulation for the development of advanced bone biomaterials. Mater. Today.

[88] He Y., Li Y., Chen G., Wei C., Zhang X., Zeng B., Yi C., Wang C., Yu D. (2020). Concentration-dependent cellular behavior and osteogenic differentiation effect induced in bone marrow mesenchymal stem cells treated with magnetic graphene oxide. J. Biomed. Mater. Res. A.

[89] Xu D., Wang C., Wu J., Fu Y., Li S., Hou W., Lin L., Li P., Yu D., Zhao W. (2022). Effects of Low-Concentration Graphene Oxide Quantum Dots on Improving the Proliferation and Differentiation Ability of Bone Marrow Mesenchymal Stem Cells through the Wnt/β-Catenin Signaling Pathway. ACS Omega.

[90] Yang X., Zhao Q., Chen J., Liu J., Lin J., Lu J., Li W., Yu D., Zhao W. (2021). Graphene Oxide Quantum Dots Promote Osteogenic Differentiation of Stem Cells from Human Exfoliated Deciduous Teeth via the Wnt/β-Catenin Signaling Pathway. Stem Cells Int..

[91] Zhao M., Dai Y., Li X., Li Y., Zhang Y., Wu H., Wen Z., Dai C. (2018). Evaluation of long-term biocompatibility and osteogenic differentiation of graphene nanosheet doped calcium phosphate-chitosan AZ91D composites. Mater. Sci. Eng. C Mater. Biol. Appl..

[92] Chen Y., Zheng Z., Zhou R., Zhang H., Chen C., Xiong Z., Liu K., Wang X. (2019). Developing a Strontium-Releasing Graphene Oxide-/Collagen-Based Organic-Inorganic Nanobiocomposite for Large Bone Defect Regeneration via MAPK Signaling Pathway. ACS Appl. Mater. Interfaces.

[93] Zhang W., Chang Q., Xu L., Li G., Yang G., Ding X., Wang X., Cui D., Jiang X. (2016). Graphene Oxide-Copper Nanocomposite-Coated Porous CaP Scaffold for Vascularized Bone Regeneration via Activation of Hif-1α. Adv. Healthc. Mater..

[94] Shim N.Y., Heo J.S. (2021). Performance of the Polydopamine-Graphene Oxide Composite Substrate in the Osteogenic Differentiation of Mouse Embryonic Stem Cells. Int. J. Mol. Sci..

[95] Zhang W., Gao R., Rong X., Zhu S., Cui Y., Liu H., Li M. (2022). Immunoporosis: Role of immune system in the pathophysiology of different types of osteoporosis. Front. Endocrinol..

[96] Zhu Y., Liang H., Liu X., Wu J., Yang C., Wong T.M., Kwan K.Y.H., Cheung K.M.C., Wu S., Yeung K.W.K. (2021). Regulation of macrophage polarization through surface topography design to facilitate implant-to-bone osteointegration. Sci. Adv..

[97] Franz S., Rammelt S., Scharnweber D., Simon J.C. (2011). Immune responses to implants—A review of the implications for the design of immunomodulatory biomaterials. Biomaterials.

[98] Liu X., Gaihre B., Park S., Li L., Dashtdar B., Astudillo Potes M.D., Terzic A., Elder B.D., Lu L. (2023). 3D-printed scaffolds with 2D hetero-nanostructures and immunomodulatory cytokines provide pro-healing microenvironment for enhanced bone regeneration. Bioact. Mater..

[99] Lv B., Wu J., Xiong Y., Xie X., Lin Z., Mi B., Liu G. (2022). Functionalized multidimensional biomaterials for bone microenvironment engineering applications: Focus on osteoimmunomodulation. Front. Bioeng. Biotech..

[100] Su J., Du Z., Xiao L., Wei F., Yang Y., Li M., Qiu Y., Liu J., Chen J., Xiao Y. (2020). Graphene oxide coated Titanium Surfaces with Osteoimmunomodulatory Role to Enhance Osteogenesis. Mater. Sci. Eng. C Mater. Biol. Appl..

[101] Zhou J., Li Y., He J., Liu L., Hu S., Guo M., Liu T., Liu J., Wang J., Guo B. (2023). ROS Scavenging Graphene-Based Hydrogel Enhances Type H Vessel Formation and Vascularized Bone Regeneration via ZEB1/Notch1 Mediation. Macromol. Biosci..

[102] Chen M., Yin J., Liang Y., Yuan S., Wang F., Song M., Wang H. (2016). Oxidative stress and immunotoxicity induced by graphene oxide in zebrafish. Aquat. Toxicol..

[103] Liu S., Xu A., Gao Y., Xie Y., Liu Z., Sun M., Mao H., Wang X. (2021). Graphene oxide exacerbates dextran sodium sulfate-induced colitis via ROS/AMPK/p53 signaling to mediate apoptosis. J. Nanobiotechnol..

[104] Mrózek O., Melounková L., Smržová D., Machálková A., Vinklárek J., Němečková Z., Komárková B., Ecorchard P. (2020). Salt-washed graphene oxide and its cytotoxicity. J. Hazard. Mater..

[105] Syama S., Mohanan P.V. (2016). Safety and biocompatibility of graphene: A new generation nanomaterial for biomedical application. Int. J. Biol. Macromol..

[106] Xu M., Zhu J., Wang F., Xiong Y., Wu Y., Wang Q., Weng J., Zhang Z., Chen W., Liu S. (2016). Improved In Vitro and In Vivo Biocompatibility of Graphene Oxide through Surface Modification: Poly(Acrylic Acid)-Functionalization is Superior to PEGylation. ACS Nano.

[107] Pelin M., Fusco L., León V., Martín C., Criado A., Sosa S., Vázquez E., Tubaro A., Prato M. (2017). Differential cytotoxic effects of graphene and graphene oxide on skin keratinocytes. Sci. Rep..

[108] Duan G., Zhang Y., Luan B., Weber J.K., Zhou R.W., Yang Z., Zhao L., Xu J., Luo J., Zhou R. (2017). Graphene-Induced Pore Formation on Cell Membranes. Sci. Rep..

[109] McCallion C., Burthem J., Rees-Unwin K., Golovanov A., Pluen A. (2016). Graphene in therapeutics delivery: Problems, solutions and future opportunities. Eur. J. Pharm. Biopharm..

